# 4,4′-Diaponeurosporene-Producing *Bacillus subtilis* Increased Mouse Resistance against *Salmonella typhimurium* Infection in a CD36-Dependent Manner

**DOI:** 10.3389/fimmu.2017.00483

**Published:** 2017-04-26

**Authors:** Haofei Liu, Wenwen Xu, Qinghua Yu, Qian Yang

**Affiliations:** ^1^College of Veterinary Medicine, Nanjing Agricultural University, Nanjing, China

**Keywords:** *Bacillus subtilis*, C30 carotenoid, CD36, mucosal defense, oral administration

## Abstract

Deficient mucosal innate immunity is a hallmark of infectious diarrhea, such as *Salmonella typhimurium* (*S*. *typhimurium*)-induced gastroenteritis. Here, we report that oral administration of a 4,4′-diaponeurosporene-producing *Bacillus subtilis* (B.s-Dia) could improve mice mucosal immunity, as showed by an increased resistance against *S. typhimurium* infection. Intragastric administration of B.s-Dia for 7 days could increase the secretion of CCL20 by intestinal epithelial cells (IECs) and then recruit more dendritic cells. Meanwhile, the number of CD8αα^+^ intraepithelial lymphocytes, which play a critical role in downregulating immune responses, was also reduced, probably as a consequence of the decrease of IEC-derived TGFβ. Further study showed that CD36 played a critical role in B.s-Dia-induced immune enhancement, as blocking CD36 signal with a specific antagonist, sulfo-N-succinimidyl oleate, led to the inability of B.s-Dia to enhance mucosal innate immunity.

## Introduction

The mucosal immune system can be regarded as the first immunological barrier encountered by microorganism and, in some cases, also serves as an infection site for many pathogens (1, 2). Deficient mucosal innate immunity is a hallmark of infectious diarrhea, such as *Salmonella typhimurium* (*S. typhimurium*)-induced gastroenteritis (3–5). Probiotics promoted mucosal immune system development and led to development of the pre-immune antibody repertoire (6).

Carotenoids, a subfamily of the isoprenoids containing more than 700 members, are currently used for food colorants in animal feeds and nutritional supplements (7). Nutrition balances the metabolic requirements with an appropriate immune function and plays a critical role in regulating pathogen persistence at mucosal barrier (8). Carotenoids as a dietary supplement can effectively promote innate immunity (9) and are important for the prevention of many diseases (9–11). Early studies demonstrating the ability of dietary carotenes to prevent infections have left open the possibility that the action of these carotenoids may be through their prior conversion to vitamin A. Subsequent studies showed that these non-provitamin A carotenoids were as active, and at times more active, than β-carotene in enhancing cell-mediated and humoral immune response in animals and humans (9). However, in spite of the multiple beneficial outcomes of carotenoid supplements, the instability and high cost make carotenoids difficult to widely apply, especially in remote area (12). Thus, an effective carotenoid and/or a suitable delivery system are urgently needed.

*Bacillus subtilis* (*B. subtilis*) is a well-known host for alkaline-fermented food and have been referred to act as a probiotic in virtue of its healthy benefit for human and animals (13, 14). Recently, *B. subtilis* as oral vaccine vehicles is particularly appealing. Accumulated evidence has shown the benefits of using the non-pathogenic, spore-forming bacterium as a non-invasive and highly thermostable, safe, and low-cost vaccine delivery system (15, 16). Considering the excellent antigen delivery function of *B. subtilis*, it might also serve as a powerful tool to deliver carotenoids.

In our previous study, we achieved the production of a C30 carotenoid, 4,4′-diaponeurosporene (Dia) in *B. subtilis* and found Dia was a potent inducer of dendritic cells (DCs) maturation *in vitro* (17). Here, the major emphasis of our study has been in developing a better understanding on the immune outcomes of the 4,4′-diaponeurosporene-producing *B. subtilis* (B.s-Dia) *in vivo*. Our results indicated that intragastric administration of B.s-Dia improves mice mucosal immunity, as showed by an increased resistance against *S. typhimurium*, which is a mode pathogen of intestinal infection. Then, we further investigated the mechanism underlying B.s-Dia-induced immunoenhancement. B.s-Dia promotes intestinal epithelial cells (IECs) to release CCL20 and increased the number of lamina propria (LP) DCs. Moreover, through specific antagonist blocking we showed that CD36 was critical in this progress.

## Results

### Dia-Treated CMT93 Cells Release Soluble Molecule to Stimulate DC Activation

**Figure 1:**
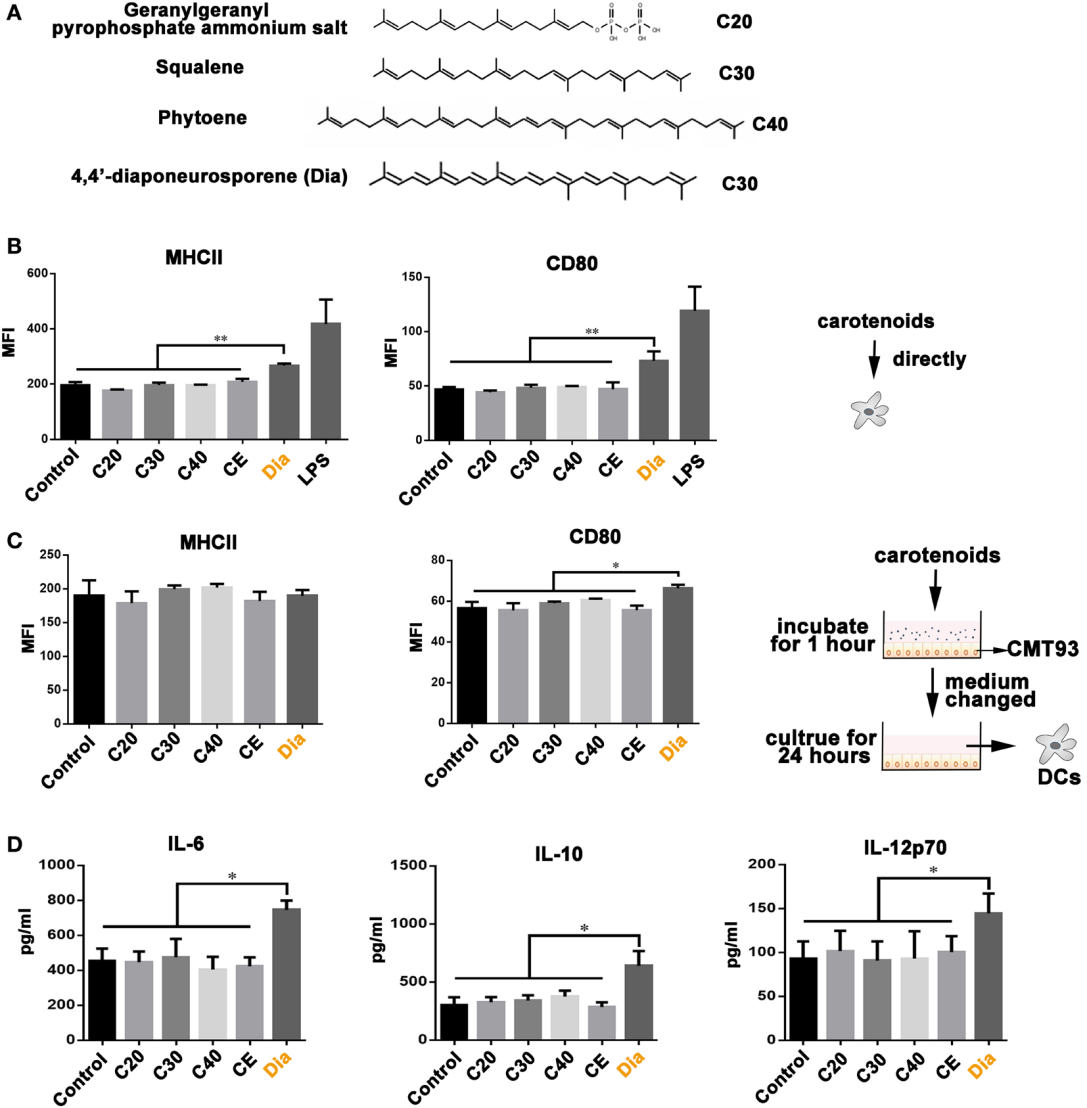
**The unique function of 4,4′-diaponeurosporene (Dia) on dendritic cells (DCs)**. **(A)** The structural representation of geranylgeranyl pyrophosphate ammonium salt, squalene, and phytoene. **(B)** DCs were treated with 1µM indicated carotenoids for 24 h, and the expression of MHCII and CD80 were analyzed by flow cytometry. CE: extraction from *Bacillus subtilis* harboring pMK3. **(C,D)** CMT93 cells were treated with different carotenoids for 1 h, and the medium containing carotenoids were removed. After washing three times, fresh medium was added and cultured for 24 h. Then, the mediums were collected to incubate DCs. After 24 h, the expression of MHCII and CD80 on DCs were analyzed by flow cytometry **(C)**, and the levels of IL-6, IL-10, and IL-12p70 in supernatants were detected by ELISA **(D)**. One representative of three similar independent experiments is shown. Asterisks indicate statistical significance *via* the one-way ANOVA test (*P < 0.05, **P < 0.01).

Our previous study revealed the different functions of Dia from β-carotene on DCs (17), bringing the possibility that the different number of carbons make them act differently. Hence, we compared the influences of carotenoids with different carbon numbers (as shown in Figure 1A) on DCs. We found only Dia could induce DCs to upregulate the expression of MHCII and CD80 (Figure 1B). The upregulation of MHCII and CD80 unlikely caused by some unknown components in Dia extraction, because DCs treated with CE (extraction from *B. subtilis* harboring pMK3) had no changes in the expression of MHCII and CD80. *In vivo*, DCs are in close contact with and are governed by IECs (18). Therefore, we tested whether carotenoids could influence DCs through stimulating epithelial cells. We incubated DCs with supernatants from mouse IEC line, CMT93, which were pretreated with different carotenoids. Only supernatant from Dia-treated CMT93 increased the expression of the cell surface activation marker CD80 on DCs (Figure 1C) and the secretion of IL-6, IL-10, and IL-12p70 (Figure 1D). The results indicated a unique function of Dia on IECs and DCs.

### B.s-Dia Improved Mouse *S. typhimurium* Resistance

**Figure 2:**
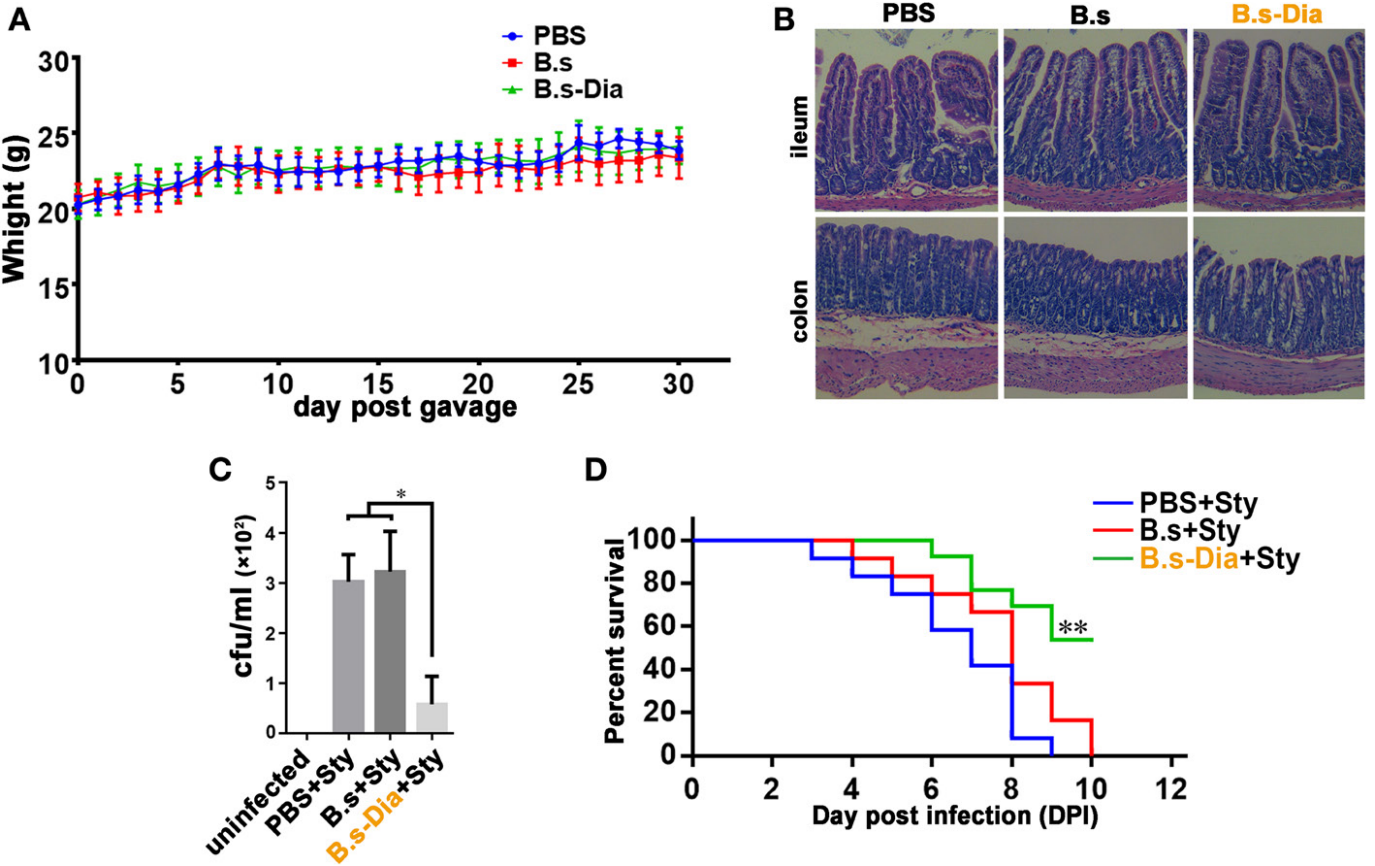
**Intragastric administration of 4,4′-diaponeurosporene-producing *Bacillus subtilis* (B.s-Dia) improved the survival of mice upon *Salmonella typhimurium* infection**. **(A)** Mice were intragastrically given 1 × 10^9^ colony forming units (cfu) B.s or B.s-Dia every day for 1 month, and the body weight was measured. **(B)** On day 30, mice were sacrificed, and the ileum and colon were removed, and paraffin sections were made and followed by hematoxylin and eosin staining. **(C,D)** Mice were given PBS or 1 × 10^9^ cfu B.s or B.s-Dia every day by gavage for 7 days. On day 8, all mice were intragastrically infected with 5 × 10^8^ cfu *S. typhimurium* (Sty). After 12 h, the number of *S. typhimurium* in mice mesenteric lymph nodes was counted by Luria–Bertani (LB) ager plate containing 500 µg/ml streptomycin **(C)**. Mouse survival rate was recorded daily for 10 days **(D)**. Survival curves were analyzed using a Kaplan–Meier survival analysis with log-rank tests. One representative of three similar independent experiments is shown (*n* = 12, **P < 0.01).

Before evaluating the immune function of B.s-Dia, we asked if administration of *B. subtilis* harboring control plasmid (B.s) or B.s-Dia were safe. To this end, mice were intragastrically given 1 × 10^9^ colony forming units (cfu) B.s or B.s-Dia every day for 1 month, and the body was weighted daily. As shown in Figure 2A, no significant changes were observed among different groups. Moreover, there were also no obvious pathological changes in ileum and colon as showed by hematoxylin and eosin staining (Figure 2B).

To test the immune function of B.s-Dia, we detected the influences of intragastric administration of B.s-Dia on mice survival when they were experimentally infected with *S. typhimurium*. Mice were intragastrically given PBS or 1 × 10^9^ cfu B.s or B.s-Dia every day for 7 days, followed by an experimentally infection of *S. typhimurium*. After 12 h, the number of *S. typhimurium* in mesenteric lymph nodes (MLN) was determined by plate count, and the rest of mice were retained for survival testing. As shown in Figure 2C, B.s-Dia administration significantly reduced the number of *S. typhimurium* diffused into MLN. Moreover, there was no survival in PBS or B.s groups on day 10 after infection, whereas 54% of mice given B.s-Dia were still alive (Figure 2D). These results indicated that intragastric administration of B.s-Dia improved mouse resistance against *S. typhimurium* infection.

### Intragastric Administration of B.s-Dia Increased the Number of LP DCs and Reduced the Proportion of CD8αα^+^ Intraepithelial Lymphocytes (IELs)

**Figure 3:**
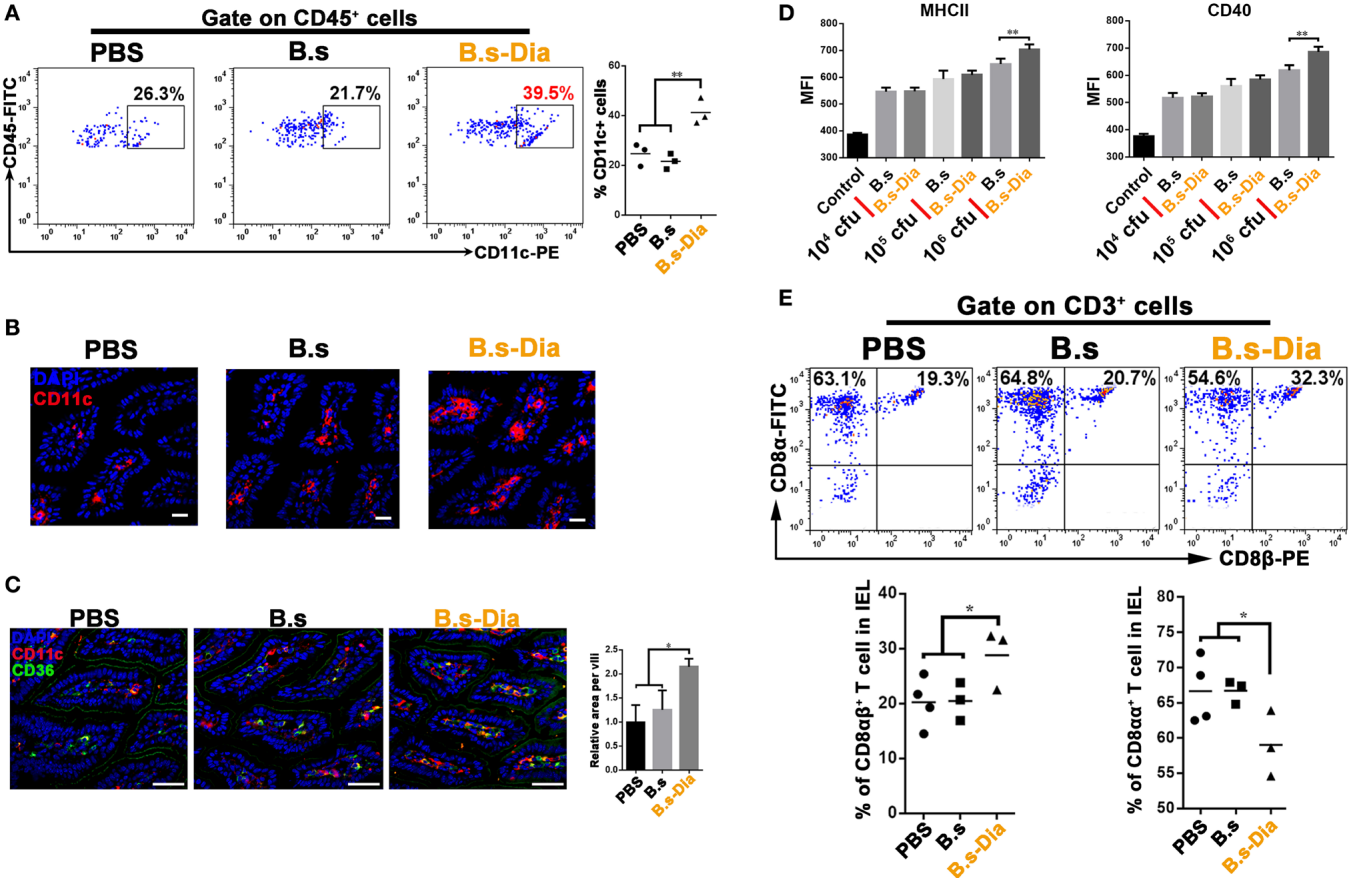
**Intragastric administration of 4,4′-diaponeurosporene-producing *Bacillus subtilis* (B.s-Dia) increased the number of lamina propria (LP) dendritic cells and reduced the proportion of CD8αα^+^ intraepithelial lymphocytes (IELs)**. Mice were intragastrically administrated of PBS, 1 × 10^9^ colony forming units B.s or B.s-Dia for 7 days. **(A)** The frequencies of CD45^+^ CD11c^+^ cells in small intestinal LP cells of the indicated mice were analyzed by flow cytometry. **(B)** Immunostaining of small intestines from the corresponding mice in **(A)** was shown. **(C)** The expression of CD36 (green) on CD11c^+^ cells (red) of the indicated mice was detected by confocal microscope. **(D)** The expression of MHCII and CD40 of CD11c^+^ cells with the indicated treatments was detected by flow cytometry. **(E)** The frequencies of IELs of the indicated mice were analyzed by flow cytometry. One representative of three similar independent experiments is shown. Asterisks indicate statistical significance *via* the one-way ANOVA test (*P < 0.05, **P < 0.01). Scale bar, 100 µm.

Dendritic cells that line the gastrointestinal tract play a key role in the establishment of both innate and adaptive immune responses (19). Gut commensal microbes shape the mucosal immune system by regulating the differentiation and expansion of DCs in LP (20, 21). To investigate the mechanisms underlying B.s-Dia-induced immune enhancement, we tested the influences of B.s-Dia on LP CD11c^+^ cells. As shown in Figures 3A–C, B.s-Dia remarkably increased the number of LP CD11c^+^ cells as well as their expression of CD36. *In vitro* study showed that B.s-Dia also had a stronger ability to induce DCs maturation compared with B.s (Figure 3D).

The IELs, which, by their immediate proximity to antigens in the gut lumen, form the front line of immune defense against invading pathogens (22). However, most of IELs express CD8α homodimer, which is a TCR repressor, leading to a limited ability to induce immune response compared to CD8αβ^+^ IELs (23). B.s-Dia gavage reduced the proportion of CD8αα^+^ IELs and correspondingly increased the proportion of CD8αβ^+^ IELs (Figure 3E). Such a change might be beneficial for reducing the threshold for the activation of immune response to invading pathogens.

### B.s-Dia Enhanced CCL20 Production but Inhibited TGFβ Production

**Figure 4:**
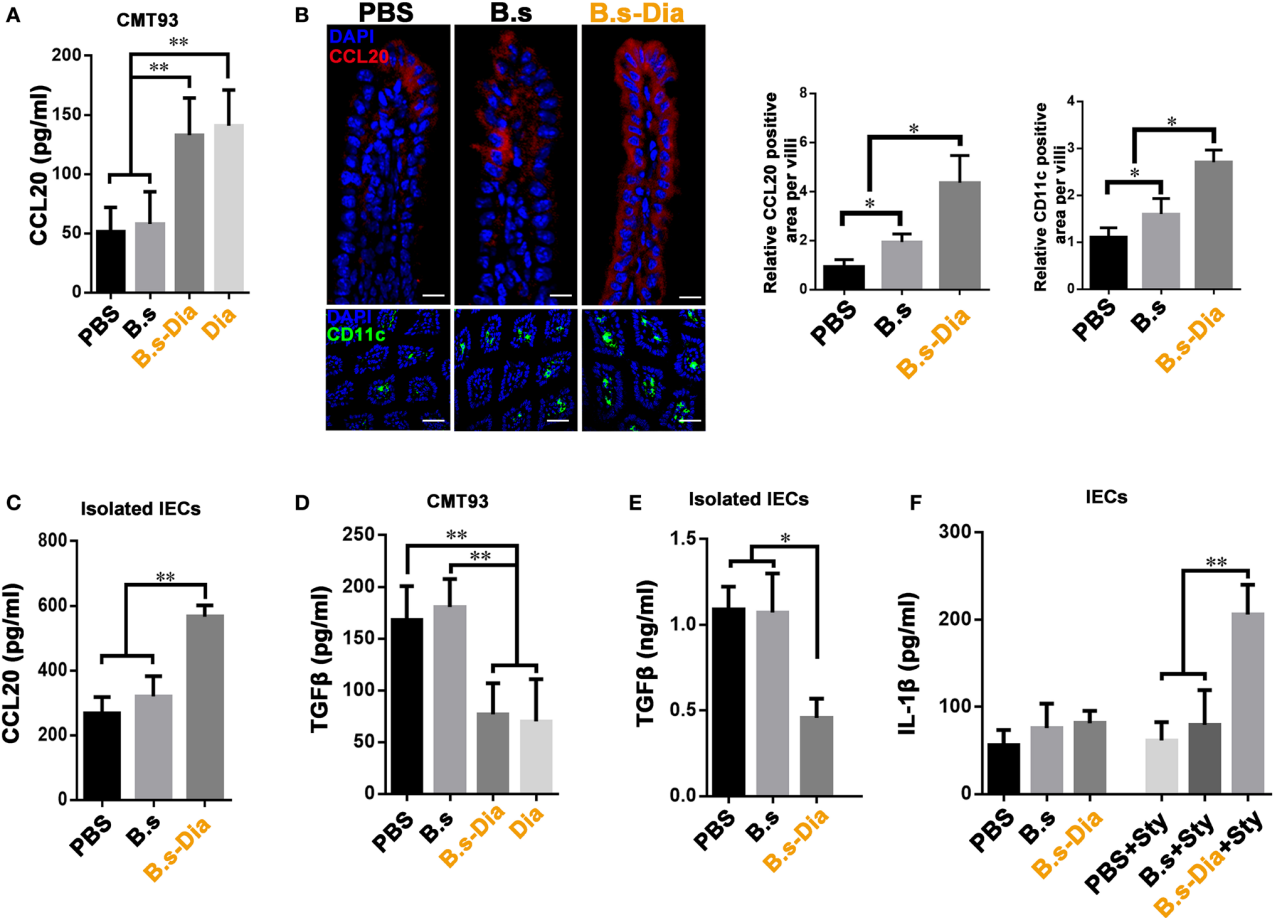
**Gavage administration of 4,4′-diaponeurosporene-producing *Bacillus subtilis* (B.s-Dia) markedly increased intestinal epithelial cell (IEC)-derived CCL20 and reduced TGFβ production**. **(A,D)** CMT93 cell was treated with 1 µM 4,4′-diaponeurosporene, 1 × 10^7^ colony forming units (cfu) B.s or B.s-Dia, the amounts of CCL20 in cultural supernatant were measured by ELISA. **(B)** Mice were intragastrically administrated of PBS, 1 × 10^9^ cfu B.s or B.s-Dia for 7 days, the expression of CCL20 (red) in IECs of the indicated mice were detected by confocal microscope. The staining of CD11c^+^ cells (green) were shown in below panels. Scale bar, 50 µm in above panel and 100 µm in the below panel. **(C,E)** The epithelial cell layer was digested as described in Section “Materials and Methods” and cultured for 6 h at 37°C. The amounts of cytokines CCL20 or TGFβ in cultural supernatant were measured by ELISA. **(F)** Mice were intragastrically administrated of PBS, 1 × 10^9^ cfu B.s or B.s-Dia for 7 days followed by *Salmonella typhimurium* gavage (Sty), and the production of IL-1β were detected as described in **(C)**. One representative of three similar independent experiments is shown. Asterisks indicate statistical significance *via* the one-way ANOVA test (**P < 0.01, *P < 0.05).

Being positioned in close proximity to a large community of commensal microbes, IECs’ function is regulated by the encountered bacteria, which could be then translated into different signals, such as secretion of CCL20 and TGFβ, to the underlying immune cells (24, 25). IEC-derived CCL20 is a critical component to recruit intestinal DCs. To investigate the influence of B.s-Dia on CCL20 production, CMT93 cells were treated with PBS, B.s, B.s-Dia, or 1 µM purified Dia for 1 h. After 24 h, the level of CCL20 in cultural supernatant was determined by ELISA. We found B.s-Dia or Dia greatly increased CCL20 secretion by CMT93 (Figure 4A). Next, we asked if B.s-Dia had a similar effect *in vivo*. As shown in Figure 4B, the production of CCL20 by IECs was significantly increased after B.s-Dia administration. To confirm this result, we also isolated IECs by EDTA digestion, after culturing for 6 h, a conformably increased expression of CCL20 was observed in IECs from B.s-Dia gavage mice (Figure 4C). Different from CCL20, the primary outcome of TGFβ signaling in the intestinal epithelium is growth inhibition and has an important tolerogenic action on immune cells (26). B.s-Dia and Dia could reduce the production of TGFβ in CMT93 cell (Figure 4D). Consistently, a greatly reduced level of TGFβ was also observed in IECs from B.s-Dia gavage mice (Figure 4E).

Stimulation and release of pro-inflammatory cytokines are essential steps for the activation of effective innate host defense. IL-1β, one of the most important pro-inflammatory cytokine, contributes to host defense against infection by augmenting antimicrobial properties of phagocytes (27, 28). B.s-Dia administration did not induce the secretion of IL-1β on steady state; however, its production was markedly increased as soon as 6 h after *S. typhimurium* gavage (Figure 4F), while B.s or PBS gavage had no influence on IL-1β production at this time point. Our results roughly indicated that B.s-Dia might have the ability to “activate” IECs, which made them “ready” for confronting *S. typhimurium* infection.

### CD36 Signaling Was Critical in B.s-Dia-Induced Immune Process

**Figure 5:**
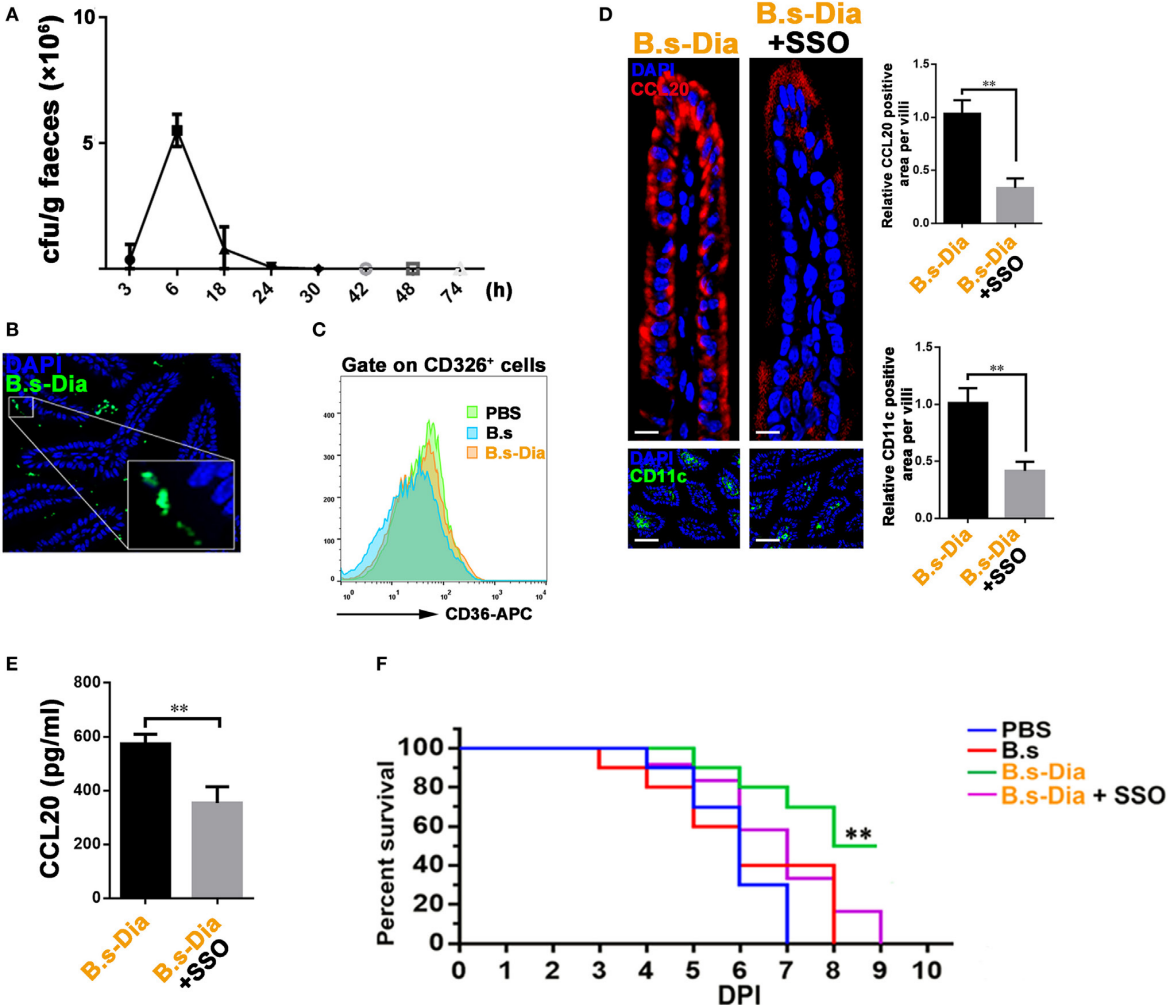
**CD36 signaling was critical in 4,4′-diaponeurosporene-producing *Bacillus subtilis* (B.s-Dia)-induced immune process**. **(A)** Mice were intragastrically administrated with 1 × 10^9^ colony forming units (cfu) B.s-Dia, and the number of B.s-Dia in mouse feces at indicated time points were detected by plate count. **(B)** Six hours after intragastrical administration of Dylight 488 labeled B.s-Dia, the distribution of B.s-Dia was detected by confocal microscope. B.s-Dia (green), DAPI (blue). **(C)** The intestinal epithelial cells (IECs) of indicated mice were digested, and the expression of CD36 on CD326^+^ IECs was measured by flow cytometry. **(D)** Mice were intragastrically administrated with 1 × 10^9^ cfu B.s-Dia or equal B.s-Dia combined with 200 µg sulfo-N-succinimidyl oleate (SSO) daily for 7 days. The expression of CCL20 (red) in IECs of the indicated mice was detected by confocal microscope. The staining of CD11c^+^ cells (green) was showed in below panels. Scale bar, 20 µm in above panel and 100 µm in the below panel. **(E)** Mice were treated as described in **(C)**. IECs were then removed by EDTA digestion and cultured at 37°C for 6 h. And, the amount of CCL20 in supernatant was detected by ELISA. **(F)** Mice were treated as described in **(D)**. On day 8, all mice were intragastrically infected with 5 × 10^8^ cfu *Salmonella typhimurium*. Mouse survival rate was recorded daily for 9 days. Survival curves were analyzed using a Kaplan–Meier survival analysis with log-rank tests, *n* = 12. Other data were analyzed *via* one-way ANOVA test (**P < 0.01, *P < 0.05). One representative of three similar independent experiments is shown.

CD36 is abundant on the apical membrane of IECs and is capable of initiating intracellular signaling cascades that activate multiple genes, such as those encoding cytokines and co-stimulatory molecules (29, 30). Moreover, CD36 involves in the absorption of many carotenoids in small intestine (31). Our previous study showed that B.s-Dia increased the expression of CD36 on DCs (17). So, we asked whether CD36 also involved in B.s-Dia–IECs interaction. Though B.s-Dia did not colonize mice intestinal tract (Figure 5A), it could reach IECs surface as showed by microscopic examination (Figure 5B). This offered opportunities for B.s-Dia to stimulate IECs. Further investigation found that, although B.s-Dia could not enhance the expression of CD36 on CD326^+^ IECs (Figure 5C), CD36 signaling indeed had a critical role in B.s-Dia-induced immune activation. Blocking CD36 signal with a specific antagonist, sulfo-N-succinimidyl oleate (SSO), remarkably reduced B.s-Dia-induced CCL20 expression, accompanied by a decreased number of DCs in LP (Figure 5D). The change of CCL20 production was also confirmed by ELISA (Figure 5E). Moreover, when mice were treated with SSO, B.s-Dia also lost the ability to enhance mouse resistance against *S. typhimurium* infection (Figure 5F). SSO alone had no influence on both *S. typhimurium* infection and IEC-derived cytokines (Figure S1 in Supplementary Material). These results together indicated the critical role of CD36 in B.s-Dia-induced immune enhancement.

## Discussion

This is the first study to explore the immunological enhancement function of a carotenoid-producing probiotics *in vivo* and provides a new idea to improve mucosal immunity. Jennifer et al. generated a β-carotene-producing variant of the probiotic *Escherichia coli* strain Nissle 1917, which had the ability to activate murine DCs *in vitro*. But, it is a pity that no *in vivo* experiments were performed to examine its immune functions (32). Compared with Nissle 1917, *B. subtilis* has many advantages, such as resistance to different environmental stresses, easily prepared and maintained, low storage costs. Here, *B. subtilis* was employed to serve as a factory for producing carotenoid and, at the same time, as a delivery system. Considering the lipid solubility of carotenoids (33), it might be easier for Dia to be distributed on the cell wall of *B. subtilis*. Indeed, in *Staphylococcus aureus*, most staphyloxanthin, a Dia analog, is cell wall bounded (34). Hence, the combination of Dia with *B. subtilis* might make it much easier for Dia to connect with enterocyte.

Epithelial cells are active participants in mucosal defense. They function as sensors that detect dangerous microbial components through pattern recognition receptors and respond by sending cytokine and chemokine signals to underlying mucosal cells, such as DCs, to trigger innate, non-specific defenses and promote adaptive immune responses (15). B.s-Dia could contact with and activate IECs. By means of expressing bacterial adhesions on surface, *B. subtilis* is capable of binding to the gut epithelium and persist longer in gastrointestinal tract (35). More researches are needed to make sure whether a prolonged retention time is a benefit for B.s-Dia to improve mucosal immune function.

CD36 is a class B scavenger receptor that binds ligands of both pathogen and self-origin, playing an important role in innate immune response (36, 37). It is clear that, on monocytes/macrophages, CD36 functions in recognizing pathogen-associated and danger-associated molecular pattern molecules that can initiate and sustain inflammatory responses (38). Previous study showed that CD36 is a phagocytic receptor for *S. aureus* (39), and expression of CD36 in human embryonic kidney (HEK) 293T cells conferred a threefold increase in binding of *S. aureus* and a twofold increase with *E. coli* over mock-transfected control cells. In fact, our previous study showed that Dia could remarkably increase the expression of CD36 on DCs, bringing up the hint that *S. aureus*, which could synthesize Dia naturally, might increase the expression of CD36 HEK293, and then further improve the uptake of bacteria. Similarly, compared with B.s, B.s-Dia-induced expression of CD36 on DCs might make it much easier for DCs to sample B.s-Dia. This might offer B.s-Dia a relatively specific action toward DCs. Except for those expressed by DCs and macrophages, CD36 is also abundantly expressed on the apical membrane of IECs and is responsible for the uptake of low density lipoprotein and carotenoids (40, 41). Recently, Cifarelli et al. showed that deficiency of CD36 impaired small intestinal barrier (42), providing the first evidence indicating the important role of CD36 in gut immunity. Now, our finding showed CD36 signaling in IECs participated in CCL20 production, which was critical in DCs recruitment, unveiling a new function of CD36 in mucosal immunity. Previous study showed that *B. subtilis* increased the secretion of CCL20 by human Caco-2 cells *in vitro* (43), but we did not observe significant increase of CCL20 production in mouse IECs after *B. subtilis* gavage. These contradictory results might be due to the species differences. However, in spite of the critical role of CD36 in Dia-related immune functions, whether CD36 is a receptor for Dia still remains to be addressed.

Intragastric administration of B.s-Dia did not influence the secretion of IL-1β by IECs on steady state but remarkably increased its production upon *S. typhimurium* infection. It seemed that B.s-Dia gavage make IECs alert to *S. typhimurium* infection. Moreover, B.s-Dia gavage also reduced the production of TGFβ and the number of CD8αα^+^ IELs. The primary outcome of TGFβ signaling in the intestinal epithelium is growth inhibition and CD8αα^+^ IELs induction (26, 44). So, the decrease of TGFβ, on the one hand, might promote epithelium growth, and on the other hand, might suppress the development of CD8αα^+^ IELs. Considering the limited ability of CD8αα^+^ IELs to induce immune response (23), its decrease might be beneficial for the defense of *S. typhimurium* infection. Taken together, oral administration of B.s-Dia could improve host defense against infections, at least in part through the positive regulation of IL-1β and the negative regulation of TGFβ–IELs axis.

**Figure 6:**
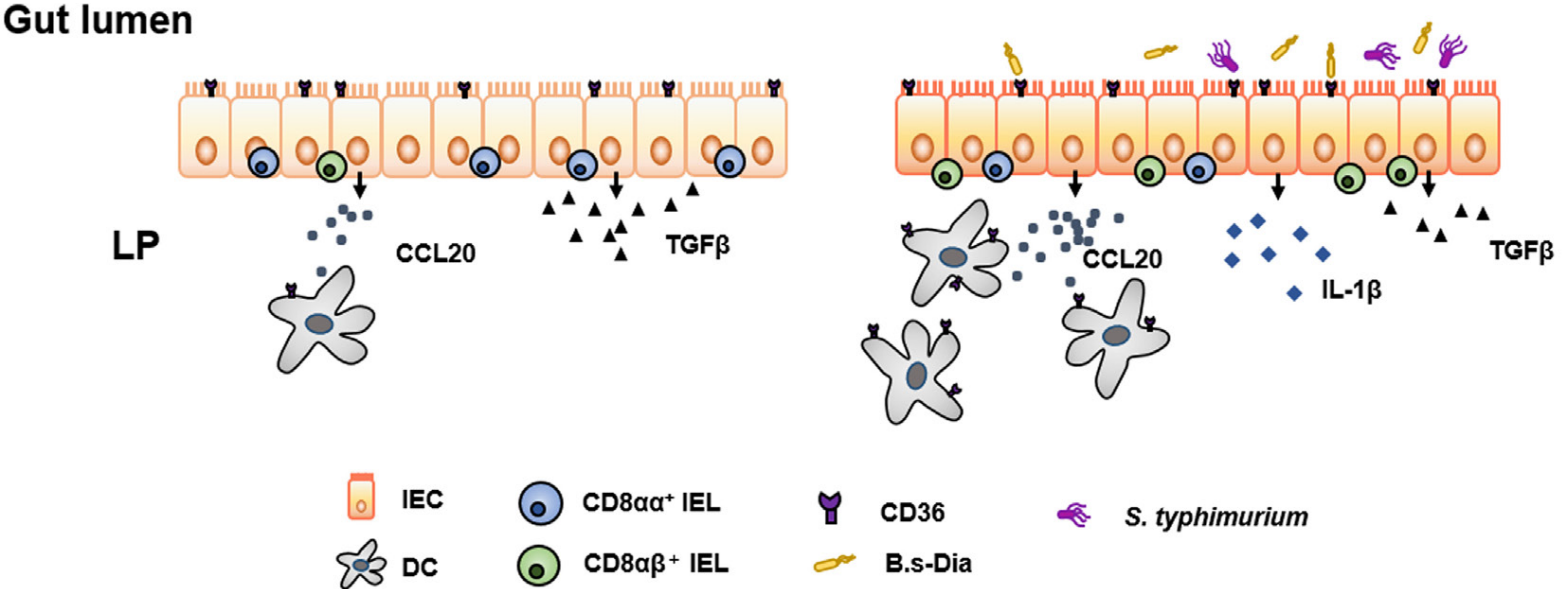
**Schematic representation of 4,4′-diaponeurosporene-producing *Bacillus subtilis* (B.s-Dia)-induced immunopotentiation**. B.s-Dia could “activate” intestinal epithelial cells (IECs) in a CD36-dependent manner. Compared with “unactivated” IECs (left panel), B.s-Dia activated IECs (right panel) upregulated the production of CCL20, recruiting more dendritic cells (DCs), and downregulated the production of TGFβ, which might be responsible for the decrease of CD8αα^+^ intraepithelial lymphocytes (IELs). When *Salmonella typhimurium* infected, activated IECs could rapidly respond and upregulated the production of IL-1β. These multiple outcomes of intragastric administration of B.s-Dia increased host defenses against *S. typhimurium*.

In conclusion, we found the B.s-Dia could increase the expression of CCL20 by IECs and recruited more DCs in a CD36-dependent manner. Moreover, it could reduce the production of TGFβ and the number of CD8αα^+^ IELs. These might make the mucosal immune system more alert to pathogenic infection and increase host defenses against *S. typhimurium* (Schematically presented in Figure 6). Though the potential off-target effects might exist, our findings uncovered a new method to improve innate mucosal immune system. More researches are needed to illustrate the immune function of B.s-Dia and its off-target effects in humans.

## Materials and Methods

### Animals, Cells, and Bacterial Strains

C57BL/6 mice, 8 weeks old, were from the Animal Research Center of Yangzhou University (Jiangsu, China). The mice  were maintained under specific pathogen-free conditions for at least 1 week before use. CMT93 cell line (ATCC CCL223) was purchased from JINIOU company (Guangzhou, China) and cultured in RPMI 1640 supplemented with 10% fetal calf serum, 100 U/ml penicillin, and 100 U/ml streptomycin. *B. subtilis* WB800 and *S. typhimurium* SL1344 were purchased form Hangzhou Biosci Biotech Company (Hangzhou, China). *S. aureus* ATCC25923 and *E. coli* DH5α were used for genetic construction. All bacteria strains were grown in Luria–Bertani (LB) broth (10 g tryptone, 5 g yeast extract, and 5 g NaCl per liter) or on LB plates fortified with 1.5% agar at 37°C. Appropriate antibiotics were included at the following concentrations: 50 µg/ml kanamycin, 100 µg/ml ampicillin, or 500 µg/ml streptomycin.

### Reagents

APC-CD11c (N418), FITC-CD40 (1C10), FITC-MHCII (M5/114.15.2), APC-CD3 (17A2), FITC-CD4 (GK1.5), PE-IL-17 (eBio17B7), PE-Foxp3 (NRRF-30), FITC-CD8α (53-6.7), PE-CD8β (H35-17.2), PE-CD326 (G8.8), APC-CD45 (MB4B4), or respective isotype controls were from eBioscience (San Diego, CA, USA). Hamster anti-mouse CD11c (N418) monoclonal antibody (mAb), rabbit anti-mouse CCL20 (MIP-3a) polyclonal antibody, rabbit anti-mouse CD36 (MF3) mAb, and SSO were from Abcam (New Territories, Hong Kong). Dylight 488-, 594-, or 647-conjugated secondary antibodies were from Jackson ImmunoResearch Laboratories (West Grove, PA, USA). Geranylgeranyl pyrophosphate ammonium salt (G6025), squalene (S3626), and phytoene (78903) were from Sigma-Aldrich (St. Louis, MO, USA).

### Obtainment of B.s-Dia

To generate B.s-Dia, we structured a *B. subtilis*–*E. coli* shuttle vector pMK3-crtMN, which conferred *B. subtilis* the ability to produce Dia after electroporation. For more details, see Ref. (10).

### Intragastric Administration with Bacteria

PBS, 1 × 10^9^ cfu *B. subtilis*, or *B. subtilis*-producing Dia (B.s-Dia) or equal B.s-Dia combined with 200 µg SSO were intragastrically administrated once a day for seven times, then sacrificed, and subsequent experiments were performed. For some mice, 5 × 10^8^ cfu *S. typhimurium* was administrated by gavage, and 6 h later, subsequent experiments were performed.

### Plate Count

Mice were given PBS or 1 × 10^9^ cfu B.s or B.s-Dia every day by gavage for 7 days. On day 8, all mice were intragastrically infected with 5 × 10^8^ cfu *S. typhimurium*. After 12 h, mouse MLN were removed under aseptic condition and cut into small pieces and grinded in 1 ml PBS. The homogenates were centrifuged at 600 × *g* for 10 min, and the supernates were plated on LB agar plates containing 500 µg/ml streptomycin and cultured at 37°C for 16 h. Then, bacterial colony on the plates was counted.

### Generation of DCs

Dendritic cells were generated as previously reported (45). Briefly, bone marrow cells of C57BL/6 mice (4 weeks old) were flushed from the tibias and femurs and cultured in complete medium (RPMI 1640 with 10% FBS, 1% streptomycin and penicillin, 10 ng/ml GM-CSF and IL-4). On day 3, the medium was gently replaced with fresh medium. On day 6, non-adherent and loosely adherent DC aggregates were harvested and subcultured overnight. On day 7, 90% or more of the CD11c^+^ non-adherent cells were used.

### *In Vitro* Culture Procedures

#### Direct Stimulation

Dendritic cells were treated with 1µM Dia, geranylgeranyl pyrophosphate ammonium salt (C20), squalene (C30), phytoene (C40) or equal volume of CE for 24 h, respectively, and the expression of MHCII and CD80 on DCs were analyzed by flow cytometry.

#### Indirect Stimulation

CMT93 cells were treated with different carotenoids for 1 h, and the medium containing carotenoids were removed. After washing three times with PBS, fresh medium was added and cultured for 24 h. Then, culture supernatants were collected to incubate DCs. Another 24 h later, the expressions of MHCII and CD80 on DCs were analyzed by flow cytometry, and the levels of IL-6, IL-10, and IL-12p70 in supernatants were detected by ELISA.

### Isolation of Lamina Propria Lymphocytes (LPLs) and IELs

Lamina propria lymphocyte isolation and intracellular cytokine staining were performed as described before (46). Briefly, mice were killed and intestines removed. After removal of residual mesenteric fat tissue and Peyer’s patches, the intestine was then cut into 1.5 cm pieces. The pieces were incubated twice in 5 ml of 5 mM EDTA in HBSS for 15–20 min at 37°C. Then, the epithelial cell layer was removed by intensive vortexing and passing through a 100 µm cell strainer. The intestine was cut in 1 mm^2^ pieces and placed in digestion solution containing 4% fetal calf serum, 0.5 mg/ml each of Collagenase D (Roche) and DNase I (Sigma), and 50 U/ml Dispase (Fisher) at 37°C for 20 min with slow rotation. After the initial 20 min, the solution was vortexed intensely and passed through a 40-µm cell strainer. The procedure was repeated a total of three times. The supernatants were combined, washed once in cold FACS buffer, resuspended in 10 ml of the 40% fraction of a 40:80 Percoll gradient, and centrifuged for 20 min at 2,500 rpm. LPLs were collected and resuspended in FACS buffer or T cell medium. The cells were used immediately for experiments.

### Surface and Intracellular Cytokine Staining

The previously harvested cells were washed twice with cold PBS and then stained with 7AAD, followed by fluorescent mAbs staining at 4°C for 0.5 h as per the manufacturer’s guidelines. After washing three times with PBS, the cells were phenotypically analyzed by FACS. For intracellular cytokine staining, the cells were incubated for 4–5 h with 50 ng/ml PMA (Sigma), 750 ng/ml ionomycin (Sigma), and 10 µg/ml brefeldin A (Invitrogen) in a cell culture incubator at 37°C. After surface staining, the cells were resuspended in fixation/permeabilization solution (BD Cytofix/Cytoperm kit—BD Pharmingen), and intracellular cytokine staining was performed as per the manufacturer’s protocol. After excluding dead cells by 7AAD, the level of IL-17 or foxp3 were analyzed with flowjo.v10.

### Immunofluorescence and Confocal Microscopy

Fixed filters were permeabilized in 0.2% Triton X-100 in PBS for 5 min. After blocking with 5% bovine serum albumin in PBS for 1 h, the filters were incubated with the primary antibodies overnight at 4°C, followed by fluorescent secondary antibodies at room temperature for 1 h. DCs were immunolabeled with Armenian hamster anti-CD11c mAb followed by Alexa Fluor 488 or 647-conjugated goat anti-Armenian hamster IgG. CD36 were labeled with rabbit anti-mouse mAb (MF3) followed by Alexa Fluor 488-conjugated goat anti-rabbit IgG. The cryosections were visualized by CLSM (LSM 710, Zeiss, Oberkochen, Germany).

### Cytokine Assays by Enzyme-Linked Immunosorbent Assay

The epithelial cell layer was digested as described above and cultured for 6 h at 37°C. The amounts of cytokines (CCL20, IL-1β, or TGFβ) in cultural supernatant were measured using enzyme-linked immunosorbent assay (eBioscience) and performed according to the manufacturer’s instructions.

### Statistical Analysis

Results were expressed as means ± SD. One-way ANOVA was employed to determine statistical differences among multiple groups, and *t*-test was employed to determine the same between two groups. *P* values <0.05 were considered significant (*P < 0.05, **P < 0.01). Survival curves were analyzed using a Kaplan–Meier survival analysis with log-rank tests.

## Ethics Statement

The animal studies were approved by the Institutional Animal Care and Use Committee of Nanjing Agricultural University and followed National Institutes of Health guidelines for the performance of animal experiments.

## Author Contributions

HL designed and performed the experiments and also wrote the manuscript; WX and Qinghua Yu contributed to the performance of the experiments; and Qian Yang designed and directed the research.

## Conflict of Interest Statement

The authors declare that the research was conducted in the absence of any commercial or financial relationships that could be construed as a potential conflict of interest.

## Supplementary Material

Figure S1 **The influences of sulfo-N-succinimidyl oleate (SSO) on *Salmonella typhimurium* infection and levels of IL-1β and TGFβ secreted by IECs**. **(A)** Mice were intragastrically administrated with PBS or 200 µg SSO daily for 7 days. On day 8, all mice were intragastrically infected with 5 × 10^8^ colony forming units (cfu) *S. typhimurium*. Mouse survival rate was recorded daily. Survival curves were analyzed using a Kaplan–Meier survival analysis with log-rank tests, *n* = 12. **(B)** Mice were intragastrically administrated of PBS, 1 × 10^9^ cfu B.s or 4,4′-diaponeurosporene-producing *Bacillus subtilis* for 7 days. The epithelial cell layer was digested and cultured for 6 h at 37°C. The amounts of cytokines IL-1β or TGFβ in cultural supernatant were measured by ELISA. Data were analyzed *via* one-way ANOVA test.
