# Supplementary figures and images for: 4,4′-Diaponeurosporene-Producing Bacillus subtilis Increased Mouse Resistance against Salmonella typhimurium Infection in a CD36-Dependent Manner
Source: Front Immunol. 2017 Apr 26;8:483. doi: 10.3389/fimmu.2017.00483 (PMC5405070; doi:10.3389/fimmu.2017.00483)

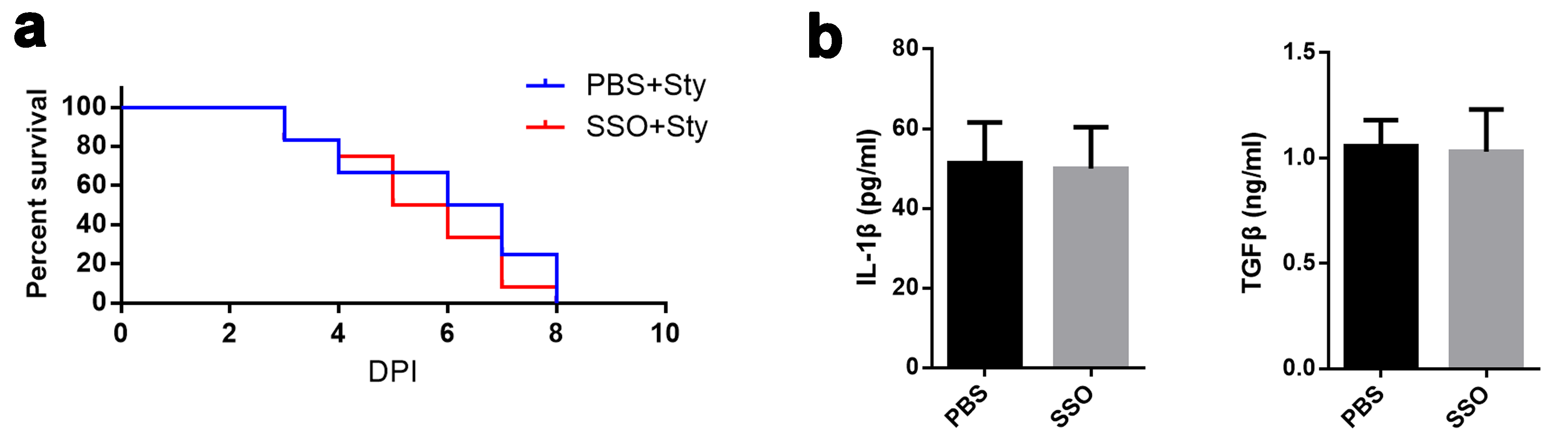

Supplement: Figure S1 — The influences of sulfo-N-succinimidyl oleate (SSO) on Salmonella typhimurium infection and levels of IL-1β and TGFβ secreted by IECs. (A) Mice were intragastrically administrated with PBS or 200 µg SSO daily for 7 days. On day 8, all mice were intragastrically infected with 5 × 10^8^ colony forming units (cfu) S. typhimurium. Mouse survival rate was recorded daily. Survival curves were analyzed using a Kaplan–Meier survival analysis with log-rank tests, n = 12. (B) Mice were intragastrically administrated of PBS, 1 × 10^9^ cfu B.s or 4,4′-diaponeurosporene-producing Bacillus subtilis for 7 days. The epithelial cell layer was digested and cultured for 6 h at 37°C. The amounts of cytokines IL-1β or TGFβ in cultural supernatant were measured by ELISA. Data were analyzed via one-way ANOVA test. [file Image_1.PNG]
